# Does Tenascin have Clinical Implications in Pathological Grade of Glioma Patients? A Systematic Meta-Analysis

**DOI:** 10.1097/MD.0000000000001330

**Published:** 2015-08-14

**Authors:** Xiangyi Kong, Wenbin Ma, Yongning Li, Yu Wang, Jian Guan, Jun Gao, Junji Wei, Yong Yao, Wei Lian, Zhiqin Xu, Wanchen Dou, Bing Xing, Zuyuan Ren, Changbao Su, Yi Yang, Renzhi Wang

**Affiliations:** From the Department of Neurosurgery, Peking Union Medical College Hospital, Chinese Academy of Medical Sciences, Beijing, P. R. China.

## Abstract

Tenascin (TN) is an extracellular oligomeric glycoprotein that participates in the adhesion of cells to extracellular matrixc (ECM). Studies have shown that the expression levels of TN are upregulated in a variety of cancers, including colon cancer, lung cancer, brain tumor, and breast cancer. However, the implications and utilities of TN in clinical grading and prognosis of glioma patients were seldom reported and the effects of its pathway are still unclear and controversial. Thus, it is essential to carry out a meta-analysis to draw a convincing conclusion.

A literature search was carried out up to April 2015. Data was collected using a purpose-designed form including glioma's WHO grade, etc. Differences were expressed as odds ratios (ORs) or standard mean differences (SMDs) with 95% confidence intervals (CIs). Galbr figure, Cochran's *Q* test, and *I*^2^ test were all performed to judge the heterogeneity between included studies. To examine the stability of the pooled results, a sensitivity analysis was performed. Potential publication bias was assessed by visual inspection of funnel plot. As this meta-analysis, as a systematic review, does not involve animal experiments or direct human trials, there is no need to conduct special ethic review and the ethical approval is not necessary.

In this meta-analysis, 8 eligible studies involving 456 patients were incorporated. Six studies with dichotomous data revealed TN overexpression in glioma tissues and/or surrounding neoplastic vessels was closely associated with high WHO grade (III + IV) (odds ratio 3.398, 95% confidence interval 1.933, 5.974; *P* = 0.000); three continuous data studies showed there were close statistical associations between TN and WHO grade (SMD −2.114, 95% CI −2.580, −1.649; *P* = 0.000) too. Sensitivity analysis indicated a statistically robust result. No publication bias was revealed.

Our meta-analysis suggests that TN expression is potentially associated with higher WHO grade of gliomas. More evidences on the basis of the evidence-based medicine are needed to prove it.

## INTRODUCTION

Gliomas are the most common primary brain tumors in adults. Standard treatment is surgery, followed by radiation therapy or combined radiation therapy and chemotherapy. According to the WHO, Gliomas are further divided into four clinical grades based on their histology and pathology.^1^ Grade I glioma is defined as relatively benign lesions. Although grades II, III, and IV gliomas usually have poorly defined margins and invade directly adjacent brain parenchyma.^2^ It should be noted that glioblastoma (Grade IV) is the most common and deadliest one, so it is very necessary to investigate an effective biomarker to predict gliomas’ WHO grade.

Granted, there have been a few markers that could serve as tools for diagnosis, prognosis, and prediction of the WHO grade of gliomas, such as 1p/19q chromosomal codeletion and IDH1/IDH2 mutations etc. But it is still commonly recognized that the prediction of clinical behavior, response to therapy, and outcome of glioma is quite challenging; hence, it is of great value and help to find more meaningful and easy-to-test biomarkers to achieve a more comprehensive and credible analysis and prediction of gliomas’ grading. We selected tenascin (TN) as such a marker mainly for 3 reasons. First, TN overexpression has been observed in many invasive and metastasizing tissues from a variety of cancers, and its roles in guiding diagnosis, prognosis, and treatment of neoplasms have been widely discussed recently, including breast cancer,^3^ bladder cancer,^4^ pancreatic cancer,^5^ ovarian cancer,^6^ and colorectal cancer,^7^ so exploring its effect on gliomas’ grading is more valid than those whose associations with malignancies have little been researched before. Second, TN, mainly as an ECM protein, is highly expressed not only in ECM, but also in cytoplasm and neoplastic vessels in malignant tissues; thus, TN's expression levels in the form of both protein and mRNA are easy to measure by virtue of immunology and histology chemistry (IHC) or polymerase chain reaction. Third, although a few studies have already confirmed that TN expression was significantly higher in gliomas than in non-neoplastic brain and correlated with gliomas’ progression,^8–10^ the precise roles of TN in WHO grading of gliomas are now still unclear and debated. In consequence, given that the meta-analysis can resolve the between-study heterogeneity, we pooled all results from published articles and systematically evaluated the expression status and implications of TN in gliomas.

## METHODS

### Search Strategy

A literature search was carried out using PubMed, Google Scholar, Medline, Cnki, and Wanfang databases up to April 2015. There were no restrictions of sources and languages. Search terms were subjected to the following: “Extracellular Matrix Metalloproteinase (EMMPRIN/TN)” or “TN,” “gliomas [MeSH],” “expression,” “grade,” etc. All references in retrieved articles were scanned to identify other potentially available reports.

### Study Selection

Two reviewers independently selected eligible studies. Disagreement between the 2 reviewers was settled by discussion with a third reviewer. Inclusion criteria were as follows: the patients were confirmed with the diagnosis of gliomas by pathologists; the main outcome of studies is WHO grade; TN expression extent or staining intensity was identified by IHC or other reliable molecular biological methods; the odds ratio OR with 95% confidence interval (CI), the mean value with SD between TN expression and WHO grading, could be obtained from articles directly or calculated based on the figures or tables given in articles, or through contacting the authors; and for the duplicate articles, only the most complete and/or the recently published one was included.

### Data Extraction

Data were extracted with a pre-designed review form. Data to be extracted were as follows: name of the first author, publication year, country, histology, study methods, WHO grade, patient number, mean ages, and the positive percentage of TN expression in the tumor specimen. Two reviewers conducted this step independently, and disagreement between them would be settled by a third reviewer.

### Quality Assessment

Two independent investigators performed the quality evaluation by browsing and grading all the eligible literatures by virtue of the quality scale put forward by the European Lung Cancer Working Party (ELCWP),^11^ including the items of scientific design, experimental methodology, generalizability, and results analysis and demonstration. The top score for each item is 10; hence, the highest score one literature could deserve is 40. Disagreement between the 2 reviewers would be grappled with by a third reviewer. The eventual results are presented in the form of the percentage of the maximum of achievable scores. Consequently, the higher the total scores are, the more desirable the quality is.

### Data Synthesis and Analysis

Differences were expressed as ORs or standard mean differences (SMDs) with 95% CIs. Galbr figure, Cochran's *Q* test, and *I*^2^ test (variation in OR attributable to heterogeneity) were all performed to judge the heterogeneity between included studies.^12,13^ In Galbr figure, if the circles are all distributed within the region bounded by the upper line and the lower line, it can be taken as an evidence of no significant heterogeneity. Heterogeneity was also considered to be significant at *P* < 0.05 for the *Q* statistic.^14^*I*^2^ values of 25%, 50%, and 75% were used as evidence of low, moderate, and high heterogeneity, respectively.^12^ If there was no evidence of statistical heterogeneity between studies, then a fixed-effects model was used. Otherwise, the random-effects model of DerSimonian and Laird was adopted.^14^

To examine the stability of the pooled results, a sensitivity analysis was performed by the one-at-a-time method, which meant omitting one study at a time and repeating the meta-analysis. If the omission of one study significantly changed the result, it implied that the result was sensitive to the studies included. Potential publication bias was assessed by visual inspection of the funnel plot, and an asymmetric plot suggested possible publication bias.^15^ An Egger linear regression test at the *P* < 0.05 level of significance was also performed to assess the publication bias.^16^ As there were only 8 included studies, meta-regression was not conducted.

All *P* values were 2-sided, and *P* < 0.05 was considered as statistically significant. Statistical analyses were performed with STATA 12.0 (StataCorp LP, College Station, TX) and SPSS 19.0 (SPSS Inc, Chicago, IL).

## RESULTS

### Search Results and Characteristics of Studies

**FIGURE 1:**
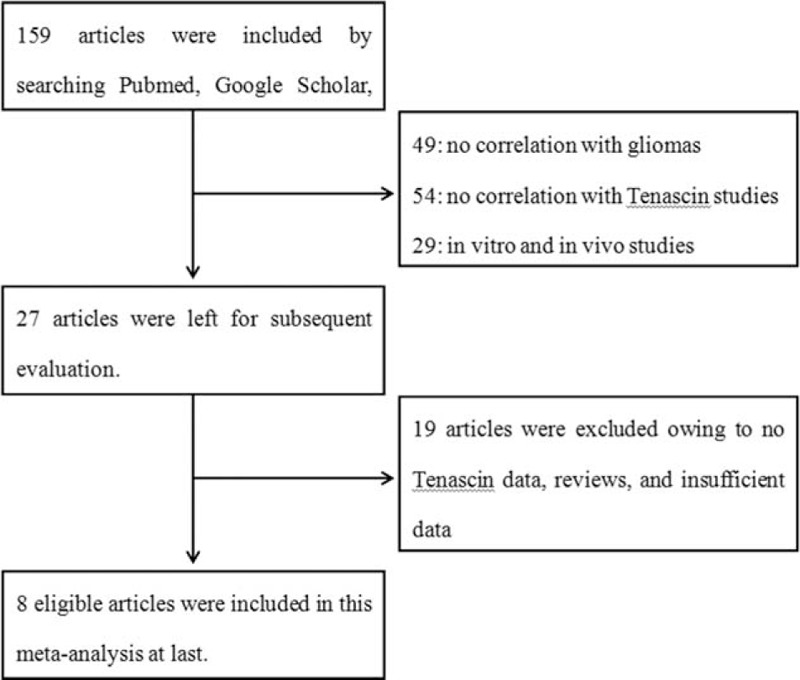
Literature search and selection of articles.

The article searches were carried out as shown in Figure 1. Initially, a literature search from multiple databases generated 159 articles. According to the title and abstract of articles, 132 articles were excluded (49 for no correlation with gliomas, 54 for no correlation with TN, and 29 for in-vitro and in-vivo studies). Subsequently, the remaining 27 articles underwent further assessment, among which 19 articles were excluded owing to no TN data, reviews, or insufficient data—the OR with 95% CI, the mean value with SD, could not be completely extracted or calculated even if related figures or tables were well-roundedly presented. At last, 8 articles that met the criteria were included,^17–24^ among which, 5 were conducted in China,^18,20,21,23,24^ 2 in Germany,^17,22^ and 1 in Japan.^19^ The general characteristics of the 8 studies were summarized in Table 1. A total of 456 patients were included and 131 cases were low-grade gliomas (I + II). The percentage of positive TN expression in dichotomous studies varies from 57.41% to 95.35%. TN protein in glioma tissues was all investigated by the method of IHC, whereas hybridization in situ was used to measure TN-mRNA. If the neoplastic cytoplasm (NC), matrix around individual tumor cells (MAC), or neoplastic vessels was stained, TN expression could be defined as positive. Two studies only measured TN expression levels in NC, and the rest also analyzed TN in the tumor environments. Thus, the standards used to value TN were not all consistent. Different studies use different cutoff values to distinguish between low and high TN expression. The details were also shown in Table 1.

**TABLE 1:**
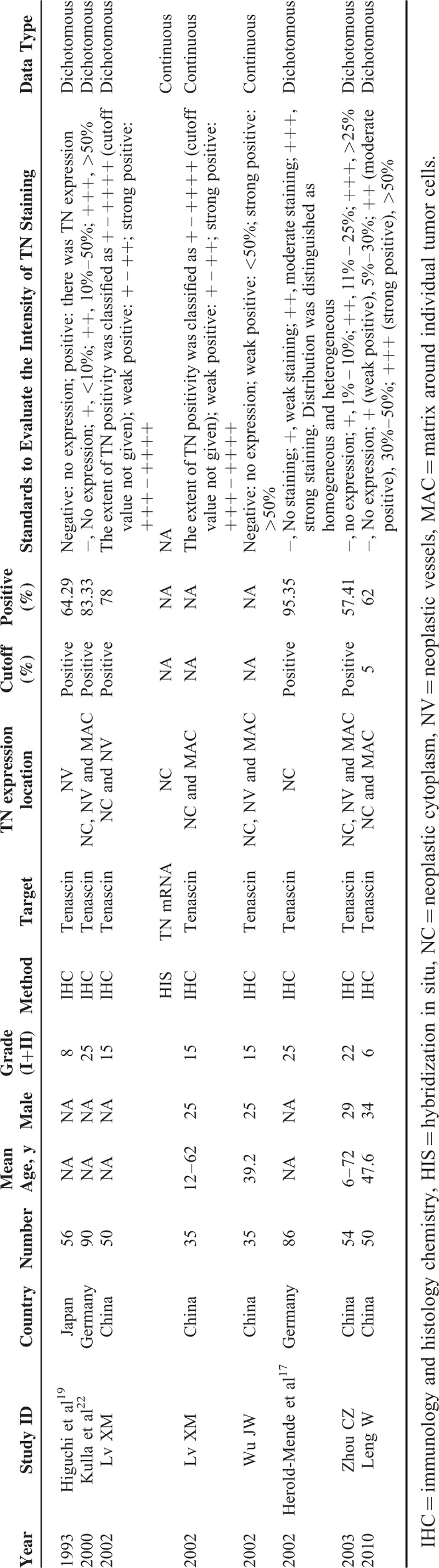
Characteristics of the 8 Included Studies

### Study Quality

We assessed the study qualities based on putative ELCWP. The detailed scores were presented in Table 2. The mean global score of the studies was 81.31%. Through all the 8 studies, results analysis obtained a high mean score of 8.34, compared with design (8.15), method (8.03), and generalizability (8.01). The mean global scores in articles with dichotomous data and continuous data were 81.312% and 80.25%, respectively, with no statistically significant differences using Student *t* test (*P* > 0.05), indicating the between study baseline characteristics may not result in potential heterogeneity.

**TABLE 2:**
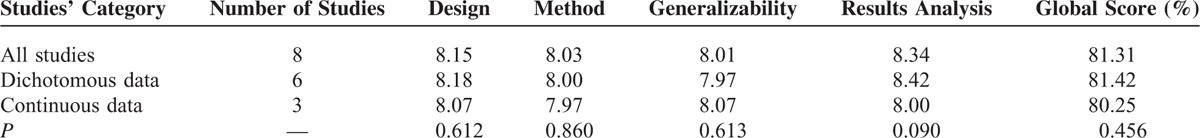
Clinical and Methodological Characteristics of 8 Included Studies

### Meta-analysis About TN and WHO Grade

**FIGURE 2:**
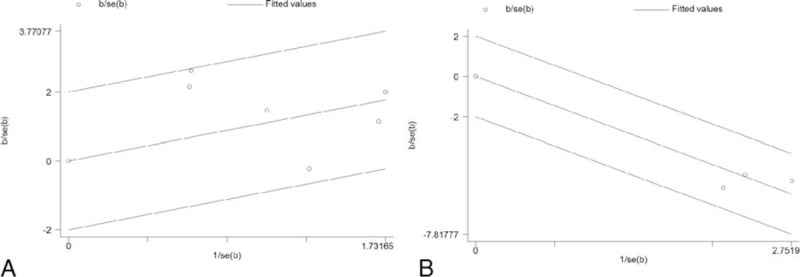
Galbr figure of included studies focusing on the correlation between tenascin (TN) and WHO grade. (A) is for 6 studies with dichotomous data, and (B) is for 3 studies with continuous data. If the circles are all distributed within the region bounded by the upper line and the lower line, it can be taken as an evidence of homogeneity. The farther from the region, the more obvious the heterogeneity is.

**FIGURE 3:**
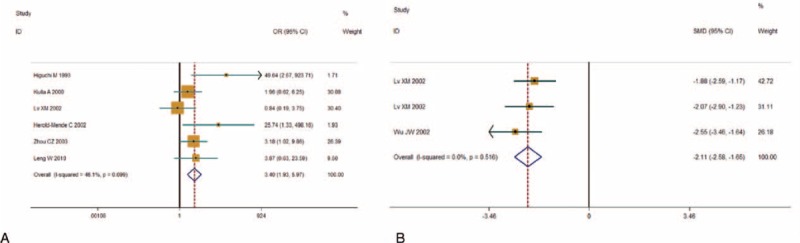
Individual and pooled effects with 95% CI about TN and WHO grade. (A) Using a fixed-effects model, 6 studies with dichotomous data showed OR 3.398, 95% CI 1.933, 5.974; *P* = 0.000, revealing an association between TN and WHO grade. (B) Using a random-effects model, 3 studies with continuous data showed SMD −2.114, 95% CI −2.580, −1.649; *P* = 0.000, thus also suggesting the association was statistically significant. CI = confidence interval, OR = odds ratio, SMD = standard mean difference, TN = tenascin.

We divided WHO grade into low (I + II) and high grade (III + IV) for the data merging. Information of WHO grading was available in 6 studies with dichotomous data^17,19,20,22–24^ and 3 studies with continuous data^18,21,23^ (Table 1). Upon dichotomous studies, from the Galbr figure (Figure 2A), we can see all points fall within the appointed region, which can be taken as evidence of homogeneity among these studies (*Q* = 9.27, d.f. = 5, *I*^2^ = 46.1%). As showed in Figure 3A, using a fixed-effects model, pooled OR revealed a significant association between TN overexpression and high WHO grade (OR 3.398, 95% CI 1.933, 5.974; *P* = 0.000) suggesting that high TN expression in postoperative glioma tissues and/or surrounding tumor environments could predict a high-grade glioma (HGG). Similarly, Galbr figure for continuous studies (Figure 2B) did not reveal heterogeneity as well (*Q* = 1.32, d.f. = 2, *I*^2^ = 0.0%). As showed in Figure 3B, also using a fixed-effects model, SMDs of the 3 studies show TN expression intensity potently correlated to gliomas’ high grading (SMD −2.114, 95% CI −2.580, −1.649; *P* = 0.000).

### Sensitivity Analysis and Publication Bias

**FIGURE 4:**
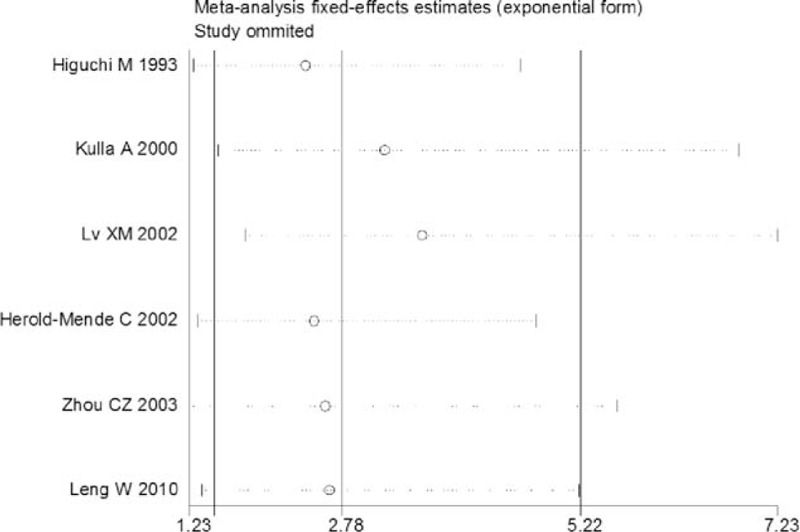
Sensitivity analysis of included articles with only IHC. Results were computed by omitting each study in turn. Meta-analysis fixed-effect estimates (exponential form) were used. The two ends of the dotted lines represent the 95% CI. CI = confidence interval, IHC = immunology and histology chemistry.

**FIGURE 5:**
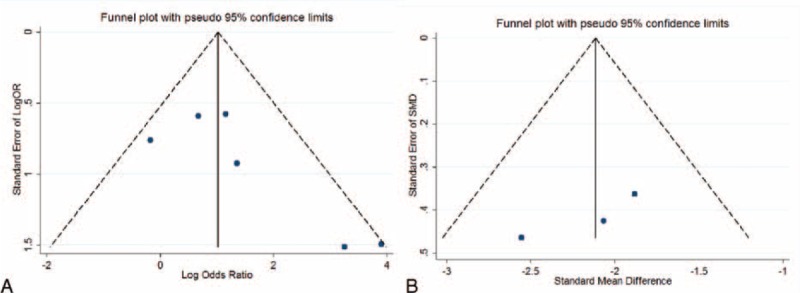
Funnel blot was designed to visualize a potential publication bias. Funnel plots’ shape of all studies did not reveal obvious evidence of asymmetry no matter for the dichotomous studies (A) or for continuous studies (B), suggesting that publication bias was also not observed among studies with pathological indicators.

Sensitivity analysis was performed to assess the influence of each individual study on the pooled OR or SMD by omitting each individual studies. The analysis results suggested that no individual studies significantly affected the pooled OR, indicating a statistically robust result (Figure 4). As there were only 3 included studies with continuous data, the sensitivity analysis was not made. In the present meta-analysis, using Begg and Egger test, less likelihood of publication bias was indicated among the 6 studies with dichotomous data (*P* = 0.094, 95% CI −0.71, 5.98), although it is difficult to draw a precise conclusion. Test results for the studies with continuous data also did not reveal bias at the statistical level (*P* = 0.247, 95% CI −37.16, 25.14). In addition, funnel plot revealed the overall symmetric distribution of all the included studies (Figure 5), indicating less likelihood of publication bias.

## DISCUSSION

TN is an extracellular oligomeric glycoprotein involved in cells’ adhesion to ECM.^25^ Consisting of several different structural domains such as EGFR-like repeats, TN is expressed in CNS during the process of embryogenesis.^26,27^ Recent research on HGGs has already revealed that TN is primarily expressed in the MAC and is inextricably with vascular proliferation, indicating that it plays a pivotal role in glimoas’ neovascularization.^8–10^ Zagzag et al^28^ have demonstrated that in human astrocytomas, TN-mRNA is highly expressed in tune with vascular proliferation. According to a study conducted by Kulla et al^22^, a larger quantity of tumor-invading macrophages or microglial cells was deposited in regions where TN was overexpressed in glioma tissues and TN is very likely to play a certain role in regulating and probably in impelling the transportation and migration of monocytes in HGGs. Moreover, as far as we know, a monoclonal antibody to TN is already being evaluated in phase 2 trials especially by physicians and scientists at Duke University. However, few studies described comprehensively the precise clinical significances of TN in glioma patients and the specific roles of TN in gliomas’ WHO grading. As such, it is still unclear whether TN can be used as a diagnostic marker. Hererin, we combined PubMed, Google Scholar, Medline, and the Chinese databases Wanfang and Cnki to systematically analyze the clinical implication of TN.

We investigated TN expression in 8 glioma studies and its association with WHO grade in 456 patients. We performed quality assessment of the eligible studies by reading and scoring each literature based on ELCWP, revealing all the studies had a relatively good quality and had no significant differences. As there was no significant heterogeneity across the studies, a fixed-effects model was chosen to determine the pooled OR and SMD estimates. Our analysis of the 6 studies with dichotomous data showed TN overexpression in glioma tissues was closely associated with high WHO grade (III + IV) (OR 3.398, 95% CI 1.933, 5.974; *P* = 0.000), and the analysis of the three studies with continuous data also reveal a significant correlation (SMD −2.114, 95% CI −2.580, −1.649; *P* = 0.000). However, eligible studies and data are insufficient after all at present, so in this circumstance, more evidences on the basis of the evidence-based medicine are needed to further prove the conclusion. In addition, as it is likely that TN expression and prognosis correlate to each other, TN may also be likely to be a potential useful prognostic and diagnostic biomarker, or an effective therapy target. But these aspects require corresponding precise studies. And checking the prognostic value of TN expression requires development of prognostic models that consider potential correlations to various tumor characteristics including WHO grading itself.

Galbr figure, Cochran's *Q* test, and *I*^2^ test were all conducted to measure the heterogeneity across all the included studies. *P* value >0.05 and/or *I*^2^ <50% indicate homogeneity. In our meta-analysis, no significant heterogeneity was revealed, so a fixed-effects model was recommended. However, several limitations of this study should be considered as many as possible: TN expression in included studies was utterly investigated in Chinese populations; the methods and criteria used to value TN expression levels were not consistent; different studies use different cutoff values to distinguish between low and high TN expression. It should be noted that differing details in the methodological factors such as primary and secondary antibody titer in these studies may influence results. However, it was very hard to conduct subgroup analyses by different antibodies to explore the potential bias of method on the pooled results. In addition, most studies did not offer complete data, although it may not affect the bias.

Publication bias is a major concern in systematic meta-analysis.^29^ Most studies are inclined to report positive outcomes, whereas the studies with negative results are often rejected. In the present study, neither Egger and Begger *P* value test nor funnel plot indicated publication bias statistically. Nevertheless, what is worth noting here is that the languages of published eligible literatures in this meta-analysis were limited to English and Chinese, which may cause publication bias because of absence of other language studies that met our inclusion criteria.

In conclusion, the present meta-analysis suggested that TN is potentially associated with high WHO grade of glioma patients. Most importantly, pathological TN detection would bring a new insight into accurate prediction and early regimen of patients undergoing surgical resection. However, to get a more determinant conclusion, more evidences on the basis of the evidence-based medicine are needed to prove the correlation.
